# Mesenchymal Stem Cell‐Conditioned Media‐Derived Exosomes Loaded With *Grifola frondosa* Extract Inhibit Lung Cancer via NF‐κB and MAPK Pathway Modulation

**DOI:** 10.1002/fsn3.70802

**Published:** 2025-08-20

**Authors:** Cailin Zhu, Dong Shang

**Affiliations:** ^1^ Department of Thoracic Surgery The First Affiliated Hospital of Xi'an Jiaotong University Xi'an China; ^2^ Department of Respiratory and Critical Care Medicine The First Affiliated Hospital of Xi'an Jiaotong University Xi'an China

**Keywords:** exosomes, *Grifola frondosa*, lung cancer, mesenchymal stem cells

## Abstract

The increasing prevalence of drug resistance diminishes the efficacy of chemotherapy and other therapeutic modalities for individuals diagnosed with lung cancer. Therefore, it is imperative to develop novel treatment approaches. This study examined how mesenchymal stem cell‐derived exosomes containing extraction of *Grifola frondosa* ( *G. frondosa* ) affected lung cancer cells. Exosomes were obtained from mesenchymal stem cells and loaded with *G. frondosa* extract (extract‐exosomes) by sonication. The effects of extract‐exosomes on A549 lung cancer cells were assessed in vitro on cell viability, colony formation, mitochondrial membrane disruption, migration, apoptosis, autophagy, and cell cycle. Moreover, we examined apoptosis‐related genes mRNA expression by real‐time PCR. Finally, both NF‐κB and MAPK signaling pathways were evaluated by western blotting method. Our results indicated that exposure to extract‐exosomes significantly inhibits lung cancer cells proliferation, colony formation, and migration. Furthermore, this treatment increased mitochondrial membrane disruption, apoptosis, and autophagy in the cancer cells. Additionally, there was a notable increase in the number of the treated cancer cells at G1 and sub‐G1 phases of the cell cycle. The research findings indicate that extract‐exosomes can inhibit lung cancer cells proliferation through the induction of apoptosis and autophagy, cell cycle arrest, as well as modification of NF‐κB and MAPK pathways.

## Graphical abstract

G. frondosa extraction loaded into exosomes (Gf‐exos). The effects of Gf‐exos on viability, macrophage polarization, colony formation, mitochondrial membrane disruption, invasion, apoptosis rate, autophagy rate, and cell cycle profile in the A549 lung cancer cells in vitro. Furthermore, expression of involved genes in apoptosis and metastasis, as well as NF‐κB and MAPK signaling pathways, was evaluated by western blotting and Real‐Time PCR methods.

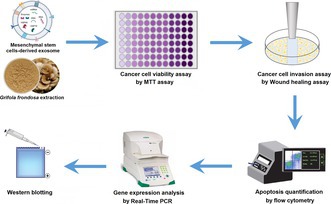

## 1 Introduction

Lung cancer is a widespread and devastating disease, affecting over 1.2 million individuals in the world. Unfortunately, the prognosis for those diagnosed with this cancer is quite bleak, with only 5%–10% achieving long‐term survival rates. The primary cause of cancer‐related deaths worldwide is non‐squamous cell lung carcinoma (NSCLC), which accounts for approximately 85% of all lung cancer diagnoses. This alarming fact highlights the urgent need to develop more effective therapeutic interventions to combat this formidable disease (Zappa and Mousa 2016; Sheikh et al. 2023). NSCLC is notably resistant to conventional therapies and often shows poor responsiveness to initial chemotherapy regimens (Sosa Iglesias et al. 2018). The use of anthracyclines in treating advanced NSCLC achieves a modest overall response rate of 30%–50%. However, severe acute and cumulative dose‐related toxicities significantly hinder therapeutic success. Additionally, the emergent use of drug resistance during treatment further complicates effective management, leading to suboptimal patient outcomes (López‐González et al. 2013).

Recent advances in understanding the molecular mechanisms of cytotoxic drug effects have shed light on potential pathways for lung cancer treatment. Increasingly, research has focused on developing novel agents targeting cellular pathways unique to cancer cells (Icard et al. 2021; Victoir et al. 2024). Exosomes, a subset of extracellular vesicles, have gained significant attention as potential vehicles for drug delivery because of their ability to transport therapeutic agents, including proteins, nucleic acids, and pharmaceuticals (Bell et al. 2016; Zhong et al. 2021). These vesicles participate in a range of physiological and pathological processes that facilitate crucial intercellular communication and material exchange. Exosomes offer several advantages, such as their natural origin, high biocompatibility, organotropic properties, and minimal immunogenicity. They can deliver bioactive molecules as therapeutic agents to different sites in various ways, playing roles in cellular metabolism, including tissue regeneration, cancer diagnostics, and immune regulation (Wang et al. 2021; Liang et al. 2021).

Evidence indicates that the edible and medicinal mushroom identified as *Grifola frondosa* ( *G. frondosa* ) has various biological properties, including anticancer, hypoglycemic, antioxidant, and immunological regulatory effects, native to Asia. A protein‐bound polysaccharide called proteoglycan, which includes β‐glucan and has an intense anti‐tumor action, is the active ingredient in *G. frondosa* (He et al. 2019). The capacity of *G. frondosa* to strengthen the immune system to suppress tumor growth, inhibit cancer metastases, and create tumor‐specific immunity has been at the center of several investigations. Furthermore, *G. frondosa* directly affects tumor cells by causing them to undergo autophagy and apoptosis (Zhao, Guo, Ma, et al. 2021; Rossi et al. 2018). *G. frondosa* has been demonstrated to inhibit various malignancies, including colon and breast tumors (Rossi et al. 2018; Roca‐Lema et al. 2019). Nevertheless, the precise methods through which *G. frondosa* impacts various forms of cancer remain elusive.

An extensive spectrum of biological activities is mainly controlled by transcription factors belonging to the nuclear factor‐kappa B (NF‐κB) family. NF‐κB's function as an oncogene in a variety of malignancies has been increasingly demonstrated by research (Zhang et al. 2017). Numerous cancers in humans have been shown to have this pathway overactivated. NF‐κB has become a promising therapeutic target in various malignancies due to recent research that has focused on clarifying its roles in immune response regulation, inflammation, and signaling pathways (Ebrahimi et al. 2024; Zhang et al. 2021). Transmitting extracellular signals from the membrane to intracellular destinations, the mitogen‐activated protein kinase (MAPK) pathway involves several biological processes (Guo et al. 2020). By converting extracellular signals, the MAPK pathway signaling may impact various biological processes in eukaryotic cells and regulate cellular functions, such as migration, differentiation, and proliferation (Guo et al. 2022). Constitutive stimulation of the MAPK pathway results in unchecked cell division and medications that induce apoptosis becoming resistant (Bahar et al. 2023).

This study explores the application of exosomes as carriers for anticancer agents. Specifically, we investigated the anticancer effects of exosomes derived from mesenchymal stem cells loaded with extracts from *G. frondosa*, a basidiomycete fungus. To gain deeper insight, the researchers investigated how this compound affects lung cancer cells by regulating two key signaling pathways: NF‐κB and MAPK. These pathways are known to influence apoptosis and autophagy.

## 2 Material and Methods

### 2.1 Exosomes Isolation

Ultracentrifugation techniques were used on the conditioned media to isolate exosomes. Initially, umbilical‐derived mesenchymal stem cells (cells/well: 1 × 10^6^ cells/well) were cultured in RPMI supplemented with 10% exosome‐depleted fetal bovine serum (FBS) for 48 h. Afterward, the medium was collected and subjected to an initial centrifugation at 800× *g* for 5 min to remove larger particles. The resulting signal was passed through a 0.22‐μm filter for further clarification. The supernatant was further purified through a series of centrifugation steps: 300× *g* for 10 min, followed by 2000× *g* for 10 min to remove ease as cells, and 10,000× *g* for 30 min at 4°C to eliminate cellular debris. To concentrate the exosomes, the medium was subjected to ultracentrifugation at 100,000× *g* for 70 min. The pellet was washed in phosphate‐buffered saline (PBS) to remove any remaining contaminants and resuspended in PBS after undergoing another ultrafiltration at 100,000× *g* for 70 min.

### 2.2 Preparation of Extract‐Loaded Exosomes

*G. frondosа* was shade‐dried and powdered using an electric blender. The powdered material was then extracted with 80% methanol for 24 h at 4°C. Subsequently, the mixture was filtered through Whatman filter paper no. 1, and the resultant solution was centrifuged to eliminate particulate matter. Methanol solvent was removed under reduced pressure using a rotary vacuum evaporator with a controlled temperature. *G. frondosа* extract was combined with exosomes in PBS and effectively loaded using the sonication technique. To facilitate sonication, an ultrasonic probe was immersed in the mixture (6 cycles; each cycle: 30 s on and 30 s off; 2‐min interval between each cycle; amplitude 20%; frequency 20 kHz). The solution was subsequently sonicated and incubated at 37°C for 30 min to facilitate the repair of the exosomal membrane. Following incubation, the mixture underwent centrifugation and was washed with PBS to isolate the exosomes, particularly those loaded with *G. frondosа* extract (extract‐exosomes). The size distribution of free and extract exosomes was evaluated by nanoparticle tracking analysis (NTA).

### 2.3 Cytotoxicity Assessment of Extract‐Loaded Exosomes

Humаn lung cancer cell line (A549) and normal human epithelial cells were seeded into 96‐well culture plates (cells/well: 1 × 10^6^ cells/well) at a concentration of 200 μL per well and allowed to adhere here for 24 h in their sрeсifiс growth medium. After this аdhesion рeriod, the сells were treated with vаrious concentrations (1, 5, 10, 20, and 40 μg/mL) of *G. frondosa* extrасtion аnd extract‐exosomes for рeriods ranging from 24 to 72 h. Post‐treatment, the cancer cells were washed with PBS. Subsequently, 200 μL of а 5 mg/mL MTT solution, рreраred in the culture medium, wаs аdded to eасh well, аnd the рlаtes were inсubаted for 4 h under stаndаrd сonditions. Fifty microliters of dimethyl sulfoxide (DMSO) was introduced to dissolve the resulting formazan crystals, followed by an additional 30‐min incubation. The optical density (OD) at 570 nm was then measured using a plate reader.

### 2.4 Migration Assay

The cancer cells were cultivated in the upper compartment of a serum‐derived Transwell apparatus (cells/well: 1 × 10^5^ cells/well), and treated with the *G. frondosa* extraction (12 μg/mL) and extract‐exosomes (8 μg/mL). The lower chamber was filled with full culture media, which included 10% fetal bovine serum (FBS) as a chemoattractant. After 24 h of incubation, the cancer cells that successfully traversed the porous membrane and reached the bottom of the compartment were fixed with a 4% paraformaldehyde solution for 15 min. Then, these migrated cells were stained with a 0.1% crystal violet solution for 10 min. We used an inverted phase‐contrast microscope to count migratory cancer cells. We accomplished this by counting stained cells in five randomly selected fields of view to determine the total number of migratory cells.

### 2.5 Colony Formation Assay

The cancer cells (cells/well: 1 × 10^5^ cells/well) were exposed to *G. frondosa* extraction (12 μg/mL) and extract‐exosomes (8 μg/mL) and cultured in a complete medium for 2 weeks. The growth medium was replenished every 72 h. After the incubation period, the resulting colonies formed by the cancer cells were immobilized using a 4% paraformaldehyde fixative solution. These colonies were then stained with a 0.1% crystal violet dye. The assessment and quantification of colony formation were performed using an inverted phase‐contrast microscope, which allowed for visualization and analysis of the stained cellular aggregates.

### 2.6 Mitochondrial Membrane Potential Assay

The cancer cells (cells/well: 1 × 10^5^ cells/well) were exposed to the *G. frondosa* extraction (12 μg/mL) and extract‐exosomes (8 μg/mL), and cultured for 48 h. After this treatment, the cells were suspended in a solution containing the fluorescent dye 3,3′‐dihexyloxacarboсyanine iodide (DiOC6). This dye is specifically used to assess the integrity of the mitochondrial membrane potential in living cells. We used fluorescence microscopy to evaluate the effect of treatment on the mitochondrial membrane potential in cancer cells. This allowed us to visualize and analyze the localization and intensity of the dye within the cell population.

### 2.7 Apoptosis Qualification Assay

The induction of apoptosis in the cancer cells (cells/well: 1 × 10^5^ cells/well) treated with *G. frondosa* extraction (12 μg/mL) and extract‐exosomes (8 μg/mL) was evaluated using 4′,6‐рhenylindole (DAPI). DAPI specifically binds to cellular nucleic acids, thereby enabling the identification of apoptotic characteristics after a 24‐h incubation period under normal culture conditions. Subsequently, the cells were fixed with a 4% paraformaldehyde solution and rendered permeable by treating them with 0.3% Triton X‐100—the DAPI staining procedure was conducted under low‐light conditions to prevent photo‐bleaching. The identification and enumeration of apoptotic cells, which were distinguished by their condensed and fragmented nuclei, were facilitated by fluorescence microscopy. This methodology permitted the visualization and counting of these cellular events.

### 2.8 Apoptosis Quantification Assay

To assess apoptosis induction in the cancer cells (cells/well: 1 × 10^5^ cells/well) treated with *G. frondosa* extraction (12 μg/mL) and extract‐exosomes (8 μg/mL), a dual fluorescence staining method was used. This involved using fluorescein isothiocyanate (FITC)‐conjugated Annexin V and propidium iodide (PI). After incubating the malignant cells with extract‐exosomes for 24 h in standard culture conditions, the cells were resuspended in 400 μL of binding buffer solution. Then, the cell suspension was stained with 5 μl of Annexin V‐FITC and 10 μl of propidium iodide (PI). After a 15‐min incubation under low‐light conditions, the stained samples were analyzed using flow cytometry. This technique allowed for the quantitative evaluation of the apoptotic cell population based on the observed differential staining patterns. Annexin V‐FITC staining identified the early stages of apoptosis, while a different staining pattern distinguished the late stages of apoptosis or necrosis.

### 2.9 Autophagy Assay

The induction of autophagy in the cancer cells (cells/well: 1 × 10^5^ cells/well) treated with *G. frondosa* extraction (12 μg/mL) and extract‐exosomes (8 μg/mL) was assessed. The mono‐dansyl cadaverine (MDC) labeling technique facilitated this assessment. After a 24‐h incubation period in standard culture conditions, the malignant cells were exposed to *G. frondosa* extraction and extract‐exosomes. They were then resuspended in a solution containing 0.05 pmol MDC. The suspension was incubated for an additional hour to allow the fluorescent MDC probe to incorporate into the autophagic vacuoles. Flow cytometric analysis was used to quantitatively evaluate autophagy by measuring the fluorescence intensity of the MDC‐labeled autophagic vacuoles and correlating it with the extent of autophagy induction in the treated cell population.

### 2.10 Cell Cycle Assay

The cell cycle distribution in the cancer cells (cells/well: 1 × 10^5^ cells/well) treated with *G. frondosa* extraction (12 μg/mL) and extract‐exosomes (8 μg/mL) was assessed by implementing propidium iodide (PI) staining. After being exposed to the *G. frondosa* extraction and extract‐exosomes and incubated for 24 h under standard culture conditions, the cells were stained with a 50 μg/mL PI solution and incubated for another hour to allow the dye to be absorbed. Flow cytometric techniques were used to analyze the different phases of the cell cycle, including G0/G1, S, and G2/M.

### 2.11 Real‐Time PCR Analysis

To evaluate mRNA expression of apoptosis‐related genes in the cancer cells (cells/well: 1 × 10^5^ cells/well) treated with *G. frondosa* extraction (12 μg/mL) and extract‐exosomes (12 μg/mL), we employed real‐time PCR. We extracted total RNA from cancer cells (cells/well: 1 × 10^5^ cells/well) using TRIzol reagent. This isolated RNA (1 μg) served as the template for cDNA synthesis. The reaction mixture for cDNA synthesis contained reverse transcriptase enzyme (1 μL), reaction buffer (4 μL), rаndom hexаmer рrimers (1 μL), dNTP mix (2 μL), and MMLV reverse transcriptase (1 μL). Following cDNA synthesis, real‐time PCR (RT‐PCR) was used to quantify the expression levels of apoptotic genes: BAX, BCL2, CASP 9, 8, and 3, BIRC5, and SMAC. The used primer sequences are presented in Table 1. The comparative 2^−ΔΔCT^ method was used to determine the relative expression of these genes, with ACTB serving as the internal control for normalization.

**TABLE 1:** The primers sequences for quantification of target genes mRNA expression.

Genes	Primers sequences	Products size
*BAX*	Forward: 5′‐CCCGAGAGGTCTTTTTCCGAG‐3′. Reverse: 5′‐CCAGCCCATGATGGTTCTGAT‐3′	155 bp
*BCL2*	Forward: 5′‐GATGGGATCGTTGCCTTATG‐3′. Reverse: 5′‐GCGGAACACTTGATTCTGG‐3′	223 bp
*CASP9*	Forward: 5′‐GCAGGCTCTGGATCTCGGC‐3′. Reverse: 5′‐GCTGCTTGCCTGTTAGTTCGC‐3′	152 bp
*CASP8*	Forward: 5′‐ACCTTGTGTCTGAGCTGGTCT‐3′. Reverse: 5′‐GCCCACTGGTATTCCTCAGGC‐3′	119 bp
*CASP3*	Forward: 5′‐ATGGTTTGAGCCTGAGCAGA‐3′. Reverse: 5′‐GGCAGCATCATCCACACATAC‐3′	122 bp
*BIRC5*	Forward: 5′‐CCCTTTCTCAAGGACCACCG‐3′. Revers: 5′‐GTTCCTCTATGGGGTCGTCA‐3′	177 bp
*SMAC*	Forward: 5′‐CAGAGGAGGAAGATGAAGTGTG‐3′. Reverse: 5′‐GCGGTTATAGAGGCCTGATCTG‐3′	196 bp
*ACTB*	Forward: 5′‐AGAGCTACGAGCTGCCTGAC‐3′. Reverse: 5′‐AGCACTGTGTTGGCGTACAG‐3′	184 bp

### 2.12 Western Blot Analysis

To evaluate the activation status of key signaling pathways (MAPK and NF‐κB) in the cancer cells (cells/well: 1 × 10^5^ cells/well) treated with *G. frondosa* extraction (12 μg/mL) and extract‐exosomes (12 μg/mL), we employed western blotting. The proteins of interest encompassed phosphorylated and non‐phosphorylated variations of JNK, p38, ERK, p65, and IκB. Total cellular proteins were isolated using a cold RIPA buffer, and 100 μg samples were loaded onto SDS‐PAGE gels to separate them based on size. The resolved proteins were subsequently transferred to PVDF membranes. To prevent non‐specific binding, the membranes were blocked using non‐fat dry milk and then probed with рrimаry аntibodies sрeсifiс to eасh tаrget рrotein (diluted 1:1000) for 24 h at 4°C. After washing, HRP‐сonjugаted seсondаry аntibodies (diluted 1:5000) were applied to amplify the signal during a 1‐h incubation at room temperature. Finally, the western blots were developed on X‐ray film, and protein band intensities were quantified using ImageJ software with β‐actin serving as the normalization control.

### 2.13 Statistical Analysis

To ensure reproducibility, all experiments were conducted independently three times. The results are reported as mean ± standard deviation (SD) to indicate the variability of the data. Statistical analysis was done using Graph Pad Prism software (version 5.0). Student's *t*‐test was used to compare the untreated control group with the treatment groups, while analysis of variance (ANOVA) was employed for comparisons involving multiple treatment groups. Bonferroni's post hoc test was used with a significance level and *p*‐value of less than 0.05 was considered as statistically significant.

## 3 Results

### 3.1 Mesenchymal Stem Cell Exosome Was Successfully Isolated and Identified

**FIGURE 1:**
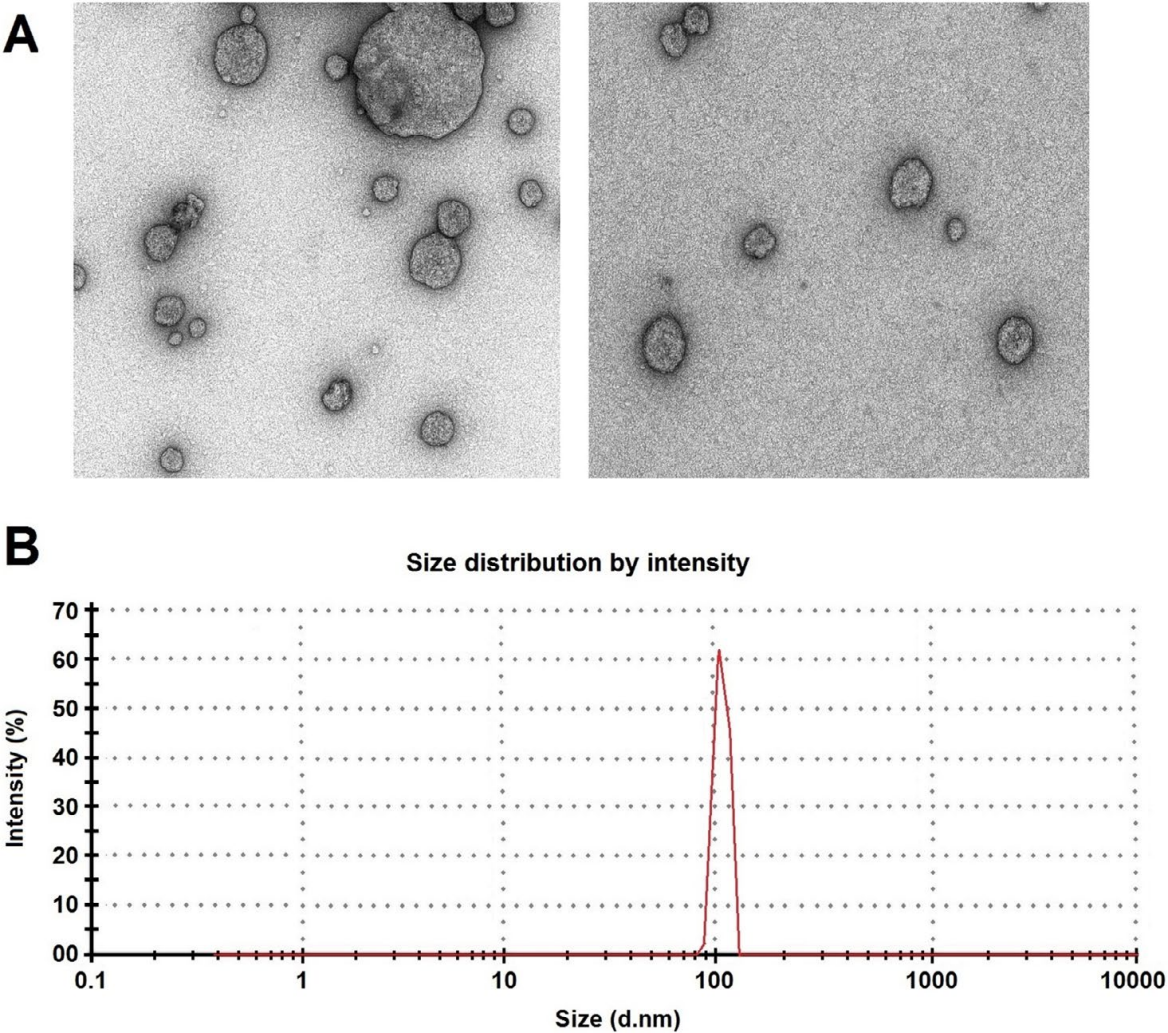
Characterization of mesenchymal stem cell‐conditioned media‐derived exosomes. (A) Transmission electron microscopic morphology of the exosomes. (B) Size distribution of the exosomes.

Exosomes were successfully isolated from stem cells by ultracentrifugation. The western blotting analysis revealed elevated expression levels of the proteins CD9 and TSG101, which are characteristic of exosomes. Their morphology and size were examined via transmission electron microscopy, revealing round, small vesicles with a double‐layer structure of approximately 100 nm in diameter. Moreover, the diameter of free exosomes was 30–100 nm (Figure 1).

### 3.2 Cancer Cell Viability Significantly Decreased After Treatment

**FIGURE 2:**
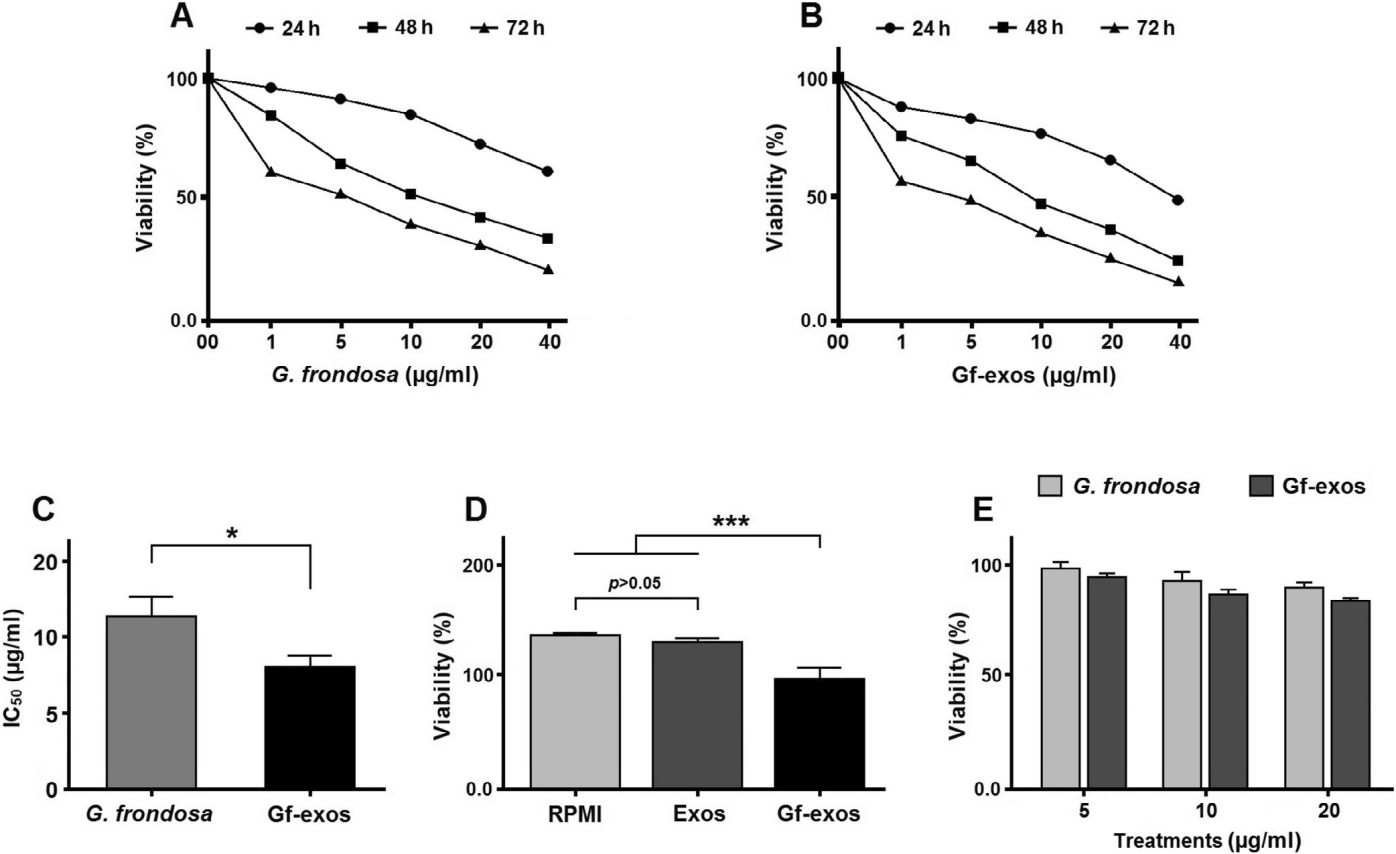
Effect of different treatments on lung cancer cells viability. (A, B) Lung cancer cells were subjected to treatments with free *Grifola frondosa* extract and exosomes loaded with *G. frondosa* extract (Gf‐exos) over varying periods: 24, 48, and 72 h. (C) The half‐maximal inhibitory concentration (IC_50_) values for both free *G. frondosa* extract and Gf‐exos were determined. (D) Naive exosomes, isolated from mesenchymal stem cells, showed no cytotoxic effect on lung cancer cells. (E) No significant toxicity was observed by *G. frondosa* extrасtion and extract‐loaded exosome on normal human epithelial cells (*p < 0.01; ***p < 0.0001).

MTT assay was employed to evaluate the effects of *G. frondosa* extrасtion and extract‐exosomes on lung cancer cell viability. The treatment indicated that extract‐exosomes significantly inhibited cancer cell viability more effectively than the free *G. frondosa* extrасtion, as demonstrated by the lower half‐maximal inhibitory concentration (IC50) (Figure 2A–C). The findings demonstrated that naive exosomes did not elicit a cytotoxic response in cancer cells, comparable to the effects observed with the RPMI culture medium (Figure 2D). Furthermore, we observed no significant toxicity by *G. frondosa* extrасtion and extract‐loaded exosome on normal human epithelial cells (Figure 2E).

### 3.3 Cancer Cell Migration Significantly Decreased After Treatment

**FIGURE 3:**
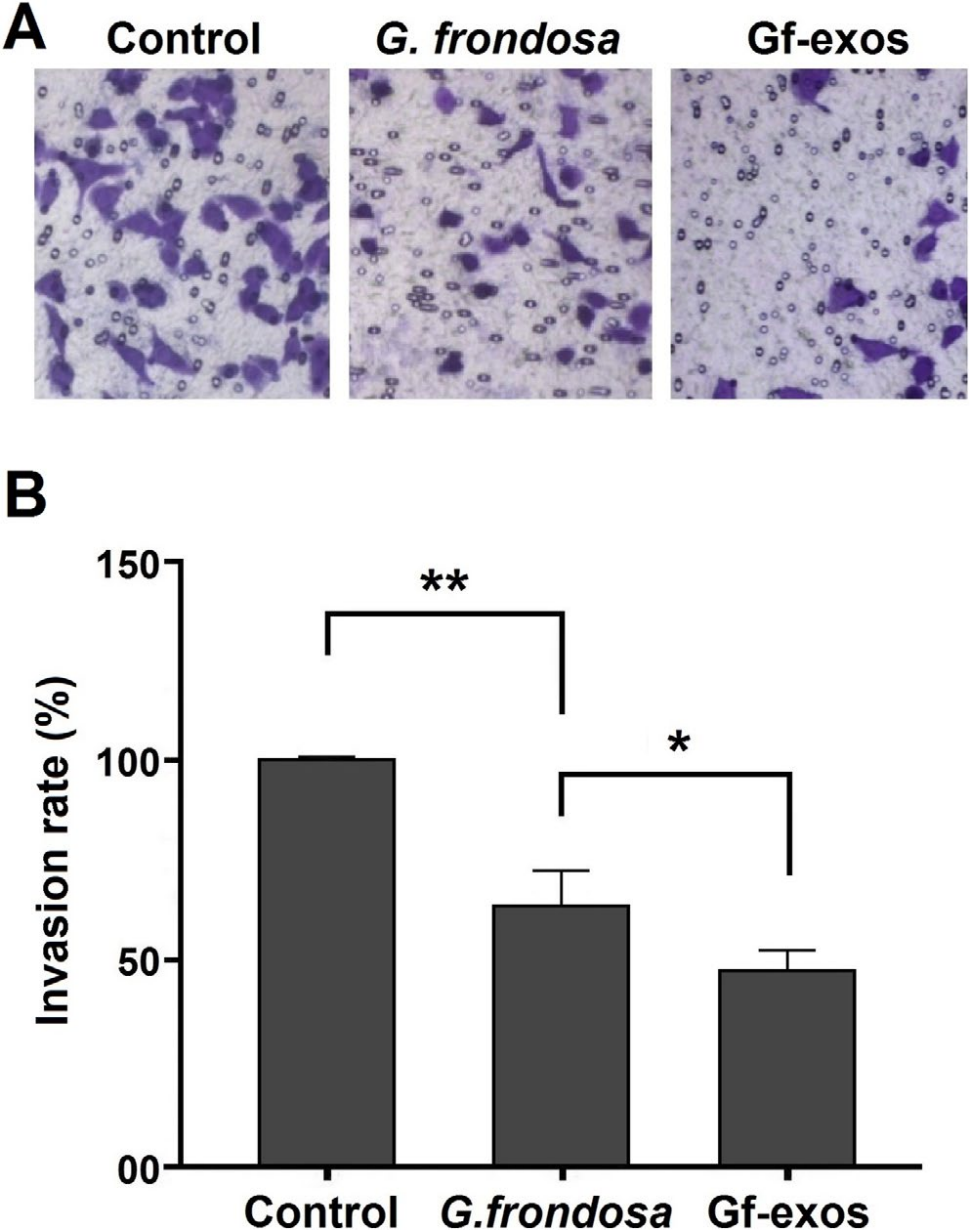
Invasion potential of lung cancer cells post‐treatment. (A) Only a few lung cancer cells were able to cross the trans‐well membrane after being treated with free *Grifola frondosa* extract and exosomes loaded with *G. frondosa* extract (Gf‐exos). (B) The invasion capabilities of lung cancer cells were significantly diminished by both the free *G. frondosa* extract and Gf‐exos treatments (*p < 0.01; **p < 0.001).

Transwell migration assay was conducted to assess the impact of free *G. frondosa* extract and extract exosomes on the migratory properties of lung cancer cells. Following treatment, there was a clear decrease in cancer cells crossing the Transwell membrane. The free *G. frondosa* extract and extract exosomes significantly inhibited cancer cell migration. However, extract exosomes exhibit a more pronounced inhibitory effect compared to the free *G. frondosa* extraction (Figure 3).

### 3.4 Cancer Cell Colony Formation Significantly Decreased After Treatment

**FIGURE 4:**
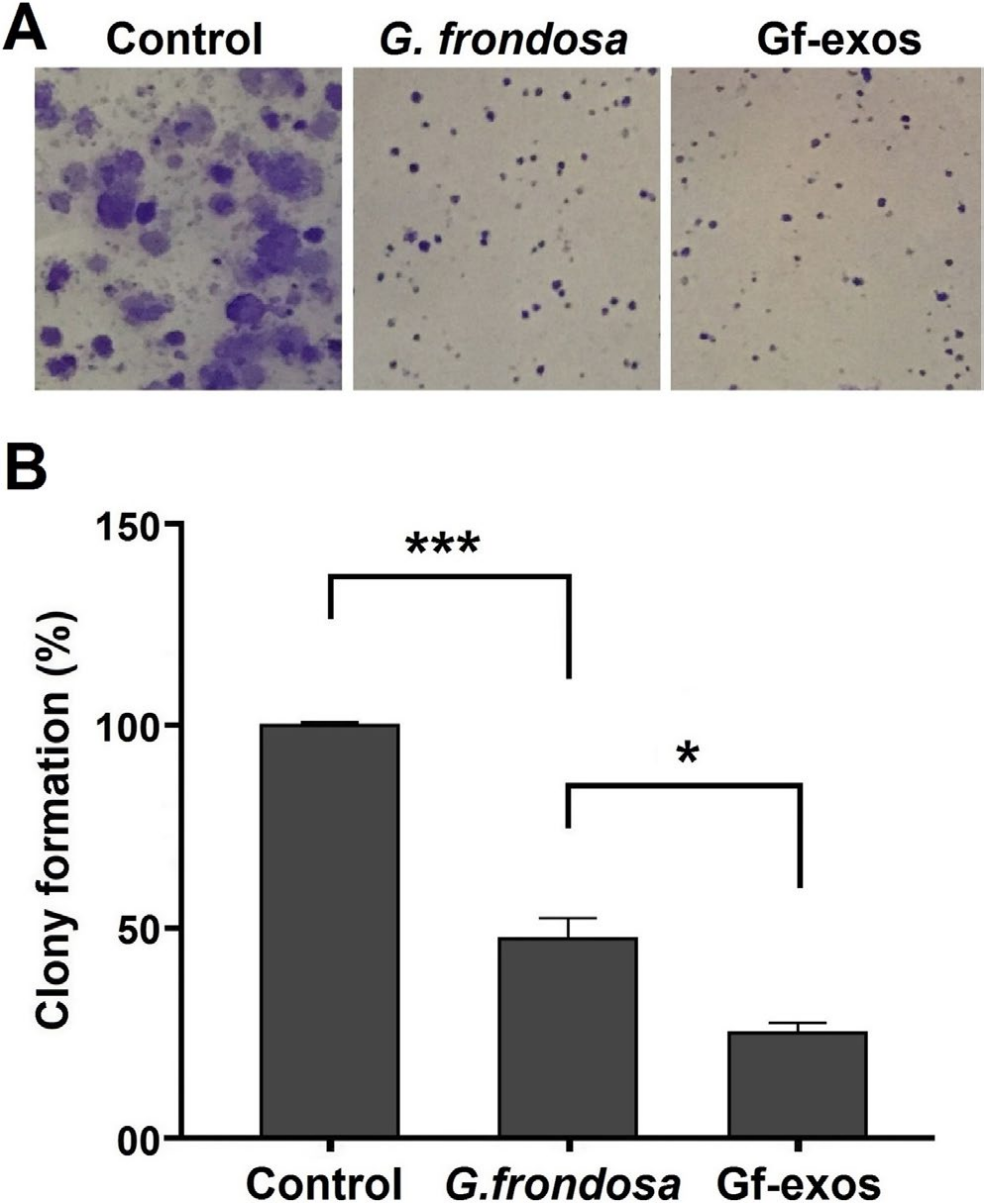
Colony forming ability of lung cancer cells post‐treatment. (A) The width and size of lung cancer cell colonies decreased after treatment with free *G. frondosa* extract and Gf‐exos. (B) Treatment with Gf‐exos significantly reduced lung cancer cells colony formation ability (*р < 0.01; ***р < 0.0001).

A colony formation assay was performed to evaluate the influence of free *G. frondosa* extract and extract‐exosomes on the capacity of lung cancer cells to establish colonies. Treatment with free *G. frondosa* extract and extract‐exosomes was observed to result in a statistically significant reduction in colony size and diameter. The number of colonies formed by lung cancer cells was significantly reduced following treatment with extract‐exosomes, exhibiting a more pronounced inhibitory effect than the free *G. frondosa* extraction (Figure 4).

### 3.5 Cancer Cells' Mitochondrial Membrane Potential Significantly Decreased After Treatment

**FIGURE 5:**
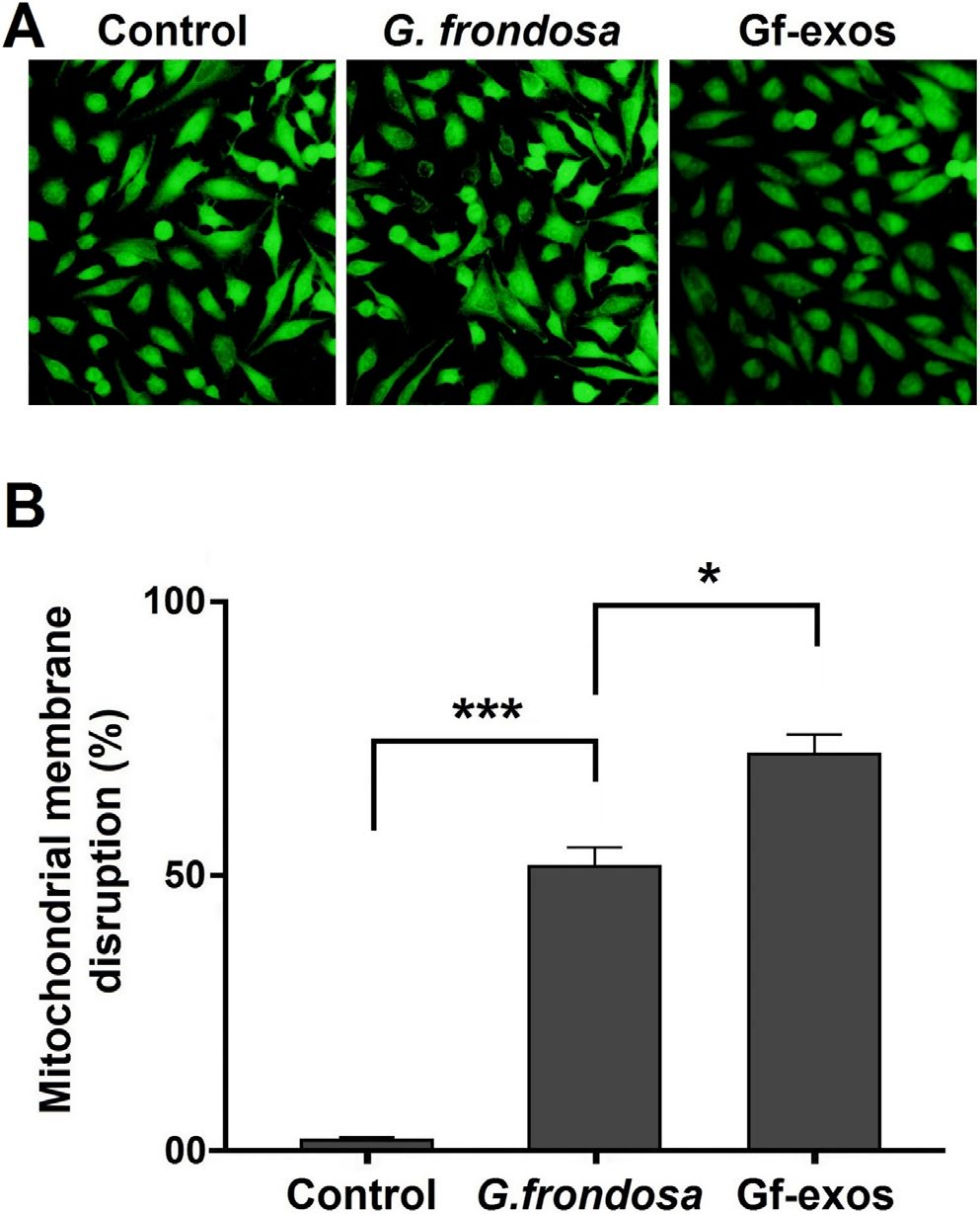
Mitochondrial membrane potential in lung cancer cells post‐treatment. (A) The mitochondrial membrane integrity in lung cancer cells is reduced after treatment with free *Grifola frondosa* extract and exosomes loaded with *G. frondosa* extract (Gf‐exos). (B) In lung cancer cells, both free *G. frondosa* extract and Gf‐exos treatments significantly increased mitochondrial membrane disruption (*p < 0.01; ***p < 0.0001).

We conducted a dedicated assay to investigate the effects of free *G. frondosa* extract and extract exosomes on mitochondrial membrane integrity in lung cancer cells. The treatment led to a noticeable decline in mitochondrial membrane integrity, as evidenced by a significant increase in the disruption rate. This indicates a decrease in mitochondrial membrane potential. Importantly, extract‐exosomes induced a higher level of disruption compared to the free *G. frondosa* extraction (Figure 5).

### 3.6 Cancer Cell Apoptosis Rate Significantly Increased After Treatment

**FIGURE 6:**
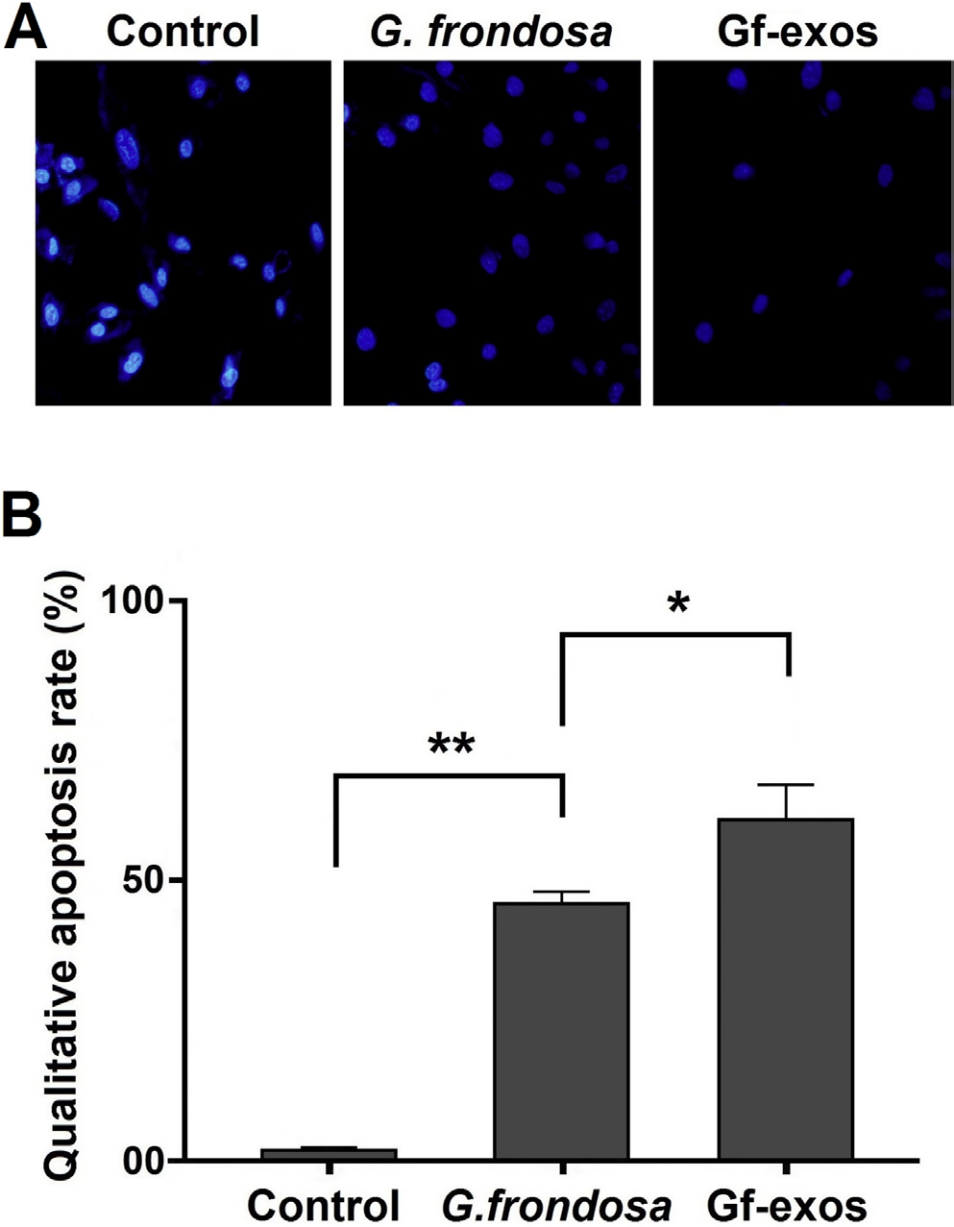
Арoptosis qualification in lung cancer cells post‐treatment. (A) After treating lung cancer cells with free *G. frondosa* extract and Gf‐exos, an increase in nuclear chromatin condensation and fragmentation was observed. (B) In lung cancer cells, treatment with free *G. frondosa* extract and Gf‐exos significantly increased apoptosis qualification (*p < 0.01; **p < 0.001).

**FIGURE 7:**
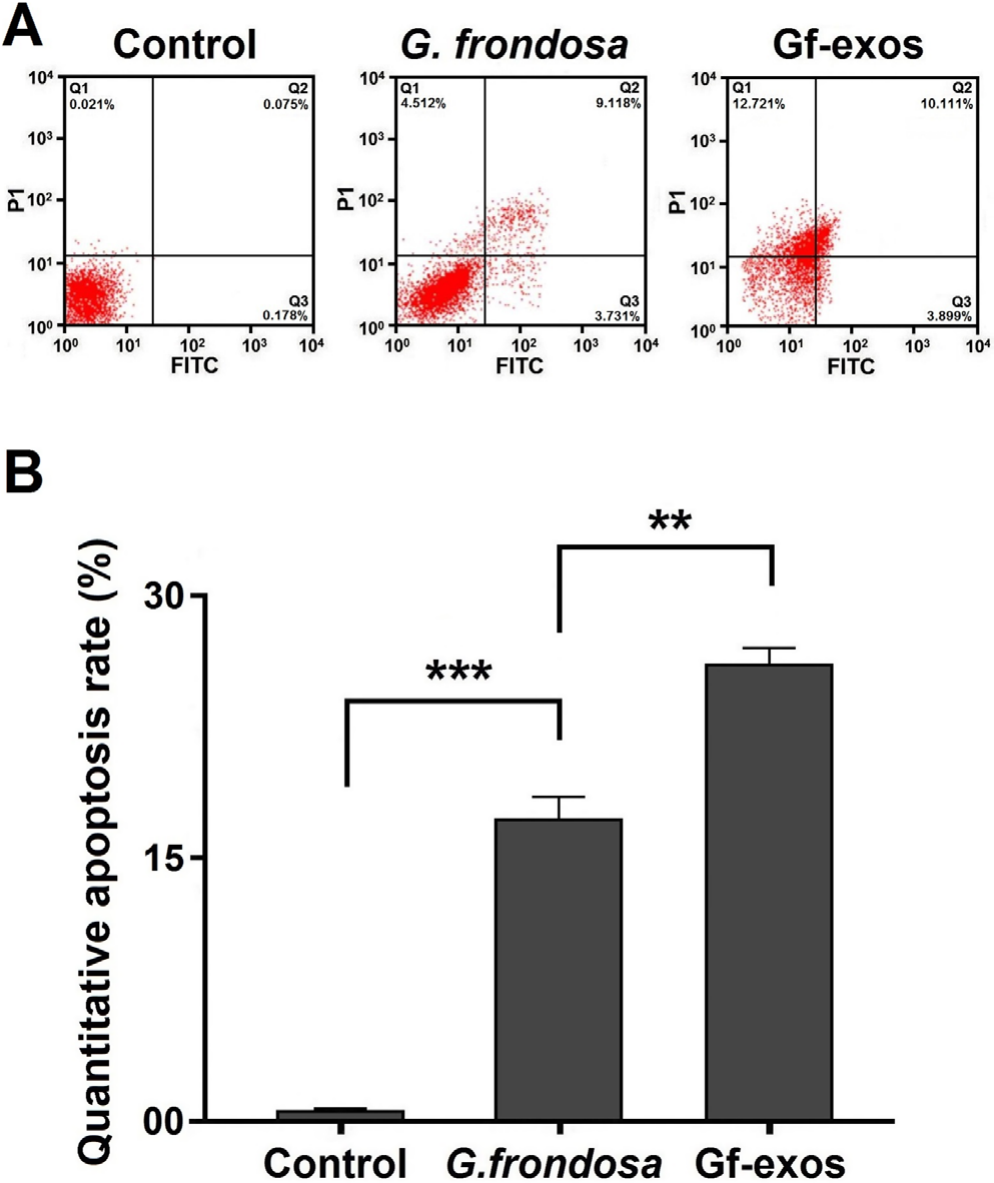
The apoptosis quantification of the lung cancer cells post‐treatment. (A) The percentage of the lung cancer cells apoptosis increased after treatment by free *G. frondosa* extraction and Gf‐exos. (B) Treatment by free *G. frondosa* extraction and Gf‐exos significantly increased apoptosis quantities in the lung cancer cells (**p < 0.01; ***p < 0.001).

DAPI and Annexin V double staining were used to evaluate the effect of free *G. frondosa* extract and extract exosomes on lung cancer cell apoptosis. Apoptosis levels significantly increased following treatment with free *G. frondosa* extract and extract exosomes. Treatment resulted in significant nuclear chromatin fragmentation and condensation, indicating apoptosis (Figure 6). Quantification of apoptosis showed a significant increase in lung cancer cells after treatment, with total apoptosis rates increasing by 17.361% and 26.731% for free *G. frondosa* extract and extract exosomes, respectively. This indicates that extract exosomes are more effective in inducing apoptosis than the free *G. frondosa* extraction (Figure 7).

### 3.7 Cancer Cells' Autophagy Rate Significantly Increased After Treatment

**FIGURE 8:**
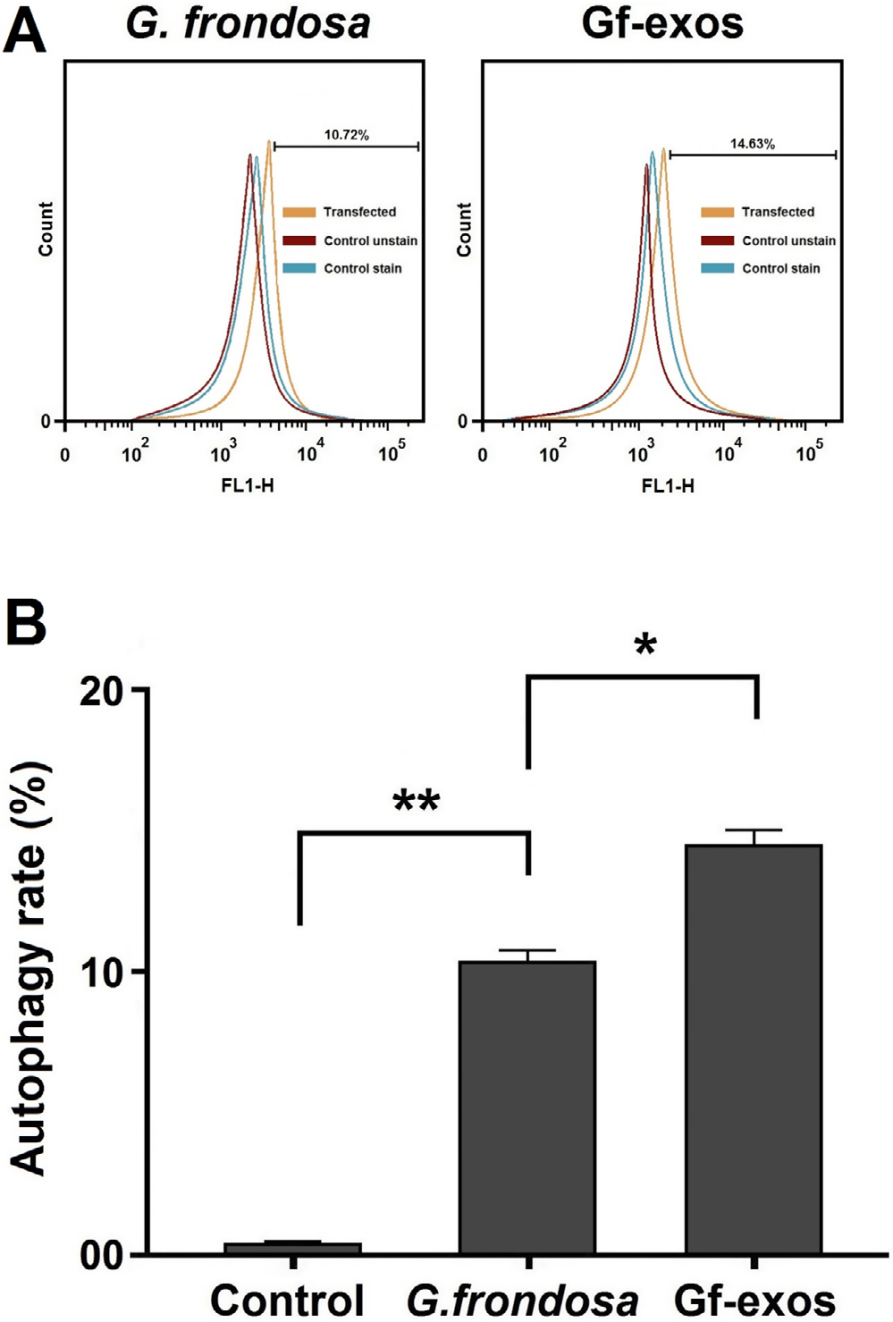
The autophagy rate of the lung cancer cells post‐treatment. (A) The percentage of autophagy in lung cancer cells increased after treatment with free *G. frondosa* extraction and Gf‐exos. (B) Treatment by free *G. frondosa* extraction and Gf‐exos significantly increased autophagy in the lung cancer cells (*p < 0.01; **p < 0.001).

The MDC labeling method assessed the impact of free *G. frondosa* extract and extract exosomes on autophagy in lung cancer cells. Treatment with both the free extract and extract exosomes significantly increased the autophagy rate in these cells. Specifically, the total autophagy percentage increased by 10.72% for the free extract and 14.63% for extract exosomes. This suggests that extract exosomes are more effective in increasing the autophagy rate than the free *G. frondosa* extract (Figure 8).

### 3.8 The Cancer Cell Cycle Is Significantly Arrested After Treatment

**FIGURE 9:**
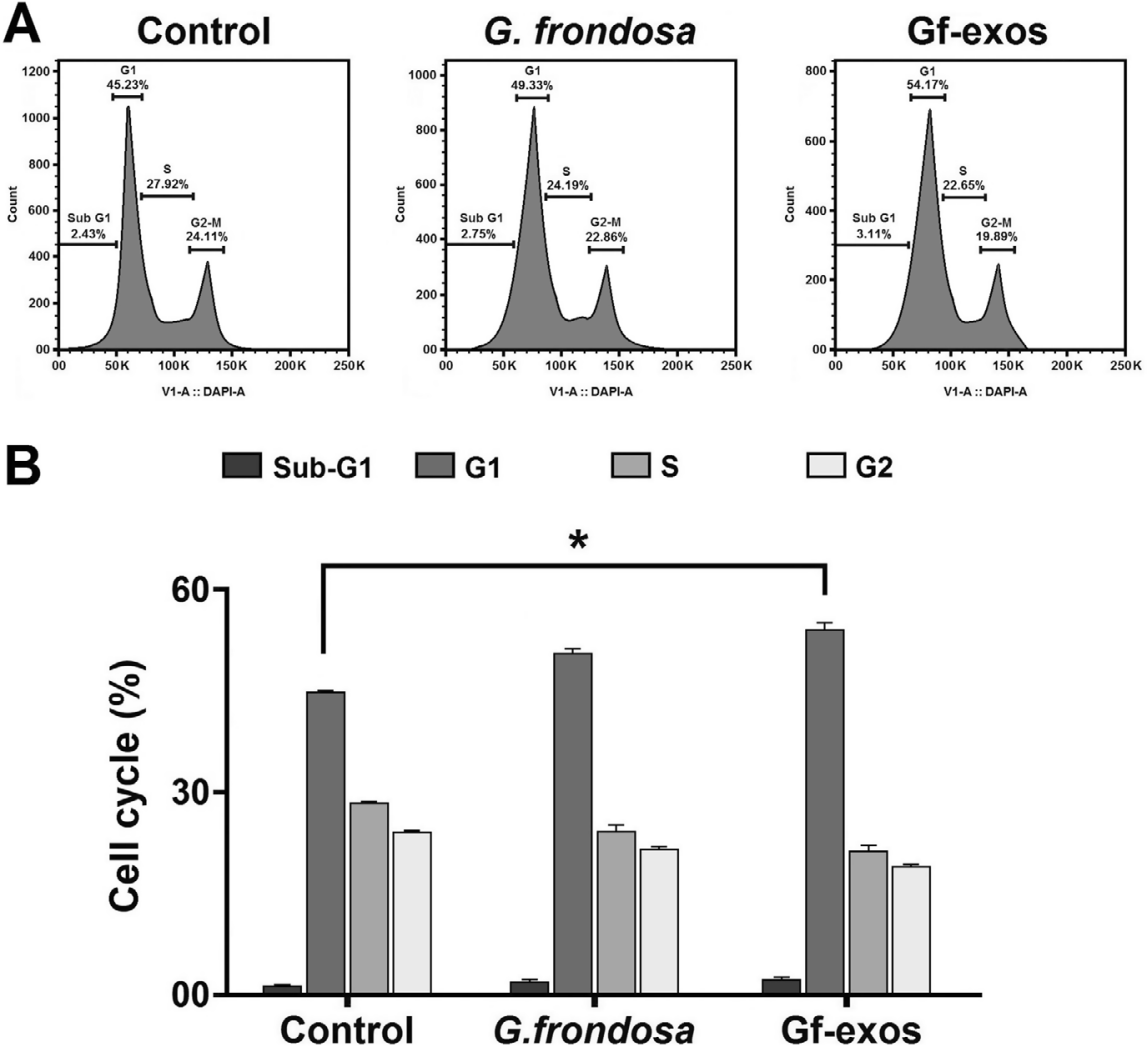
The cell cycle profile of the lung cancer cells post‐treatment. (A) After treatment with free *G. frondosa* extract and Gf‐exos, there was an increase in the percentage of lung cancer cells that underwent cell cycle arrest in the sub‐G1 and S phases (B) Treatment by free *G. frondosa* extraction and Gf‐exos significantly increased the sub‐G1 and S phases in the lung cancer cells (*p < 0.01).

Propidium iodide (PI) staining was used to assess the effect of free *G. frondosa* extract and extract exosomes on lung cancer cell cycle progression. The extract and extract‐exosomes treatment resulted in cell cycle arrest, specifically in the sub‐G1 phases. However, there was a slight increase in the G1 phase in treated cells. However, we found a non‐significant decrease in S and G2 phases in the treated cancer cells. The data presented in Figure 8 indicated a more significant cell cycle arrest induced by extract‐exosomes compared to the free *G. frondosa* extraction (Figure 9).

### 3.9 Apoptosis‐Related Gene Expression Was Significantly Modified After the Treatment

**FIGURE 10:**
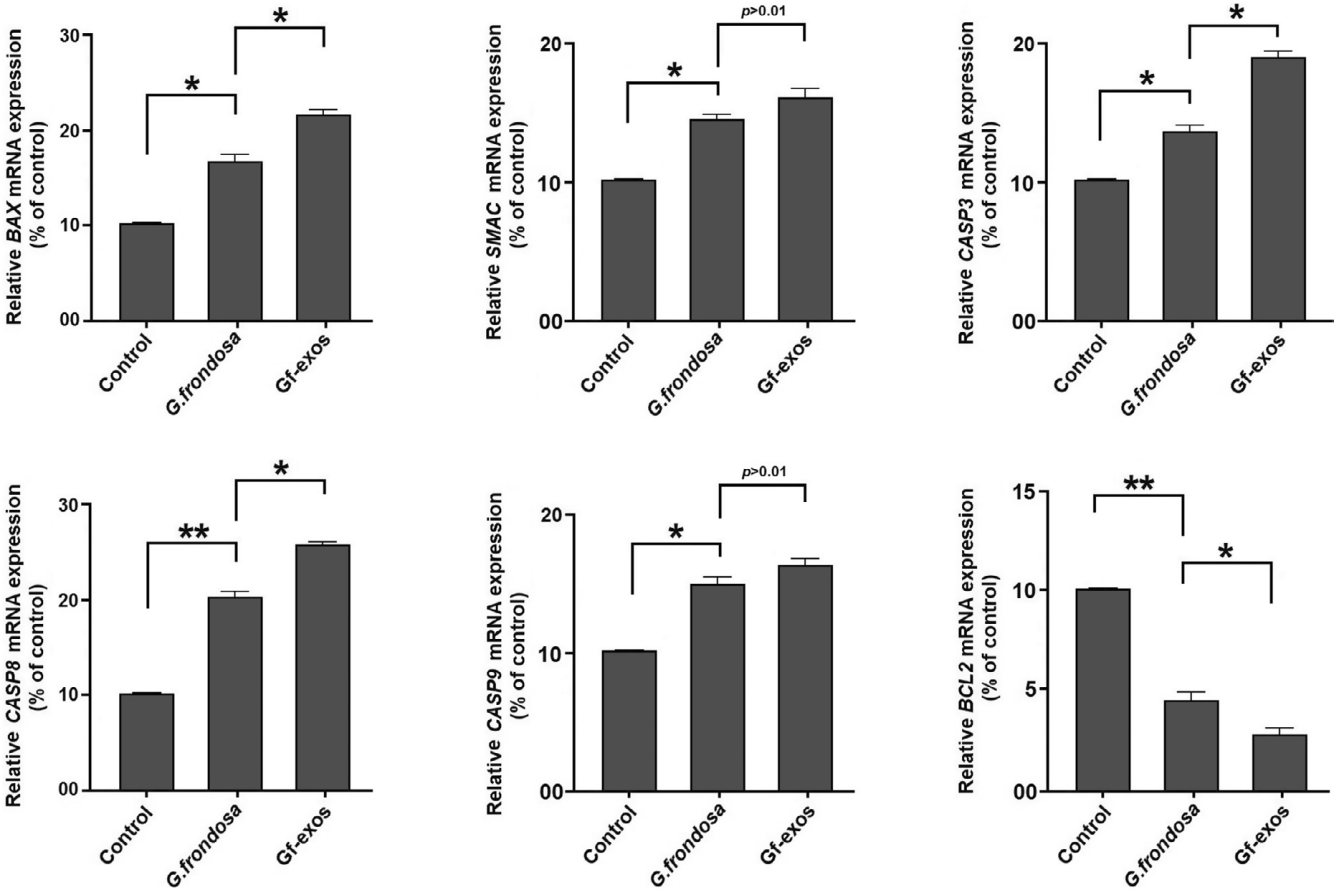
The cancer‐related genes transcribe in lung cancer cells post‐treatment. Treatment with free *G. frondosa* extraction and Gf‐exos significantly increased the transcription of pro‐apoptotic genes and significantly decreased the transcription of anti‐apoptotic and metastatic genes in lung cancer cells. The change in mRNA expression of apoptosis‐related genes in cancer cells treated with Gf‐exos was significantly greater compared to free *G. frondosa* extraction (*p < 0.01; **p < 0.001).

Real‐time PCR was used to evaluate the impact of *G. frondosa* extract and extract‐exosomes on the mRNA expression levels of apoptosis‐related genes in lung cancer cells. After treatment, there was a noticeable increase in the mRNA transcripts of pro‐apoptotic genes (BAX, CASP9, CASP8, CASP3, аnd SMAC), along with a significant decrease in the mRNA transcripts of the anti‐apoptotic gene (BCL2). These findings suggest that extract exosomes have a more pronounced effect on the expression of apoptosis‐related genes than the free *G. frondosa* extract (Figure 10).

### 3.10 MAPK and NF‐κB Signaling Pathways Were Significantly Modified After the Treatment

**FIGURE 11:**
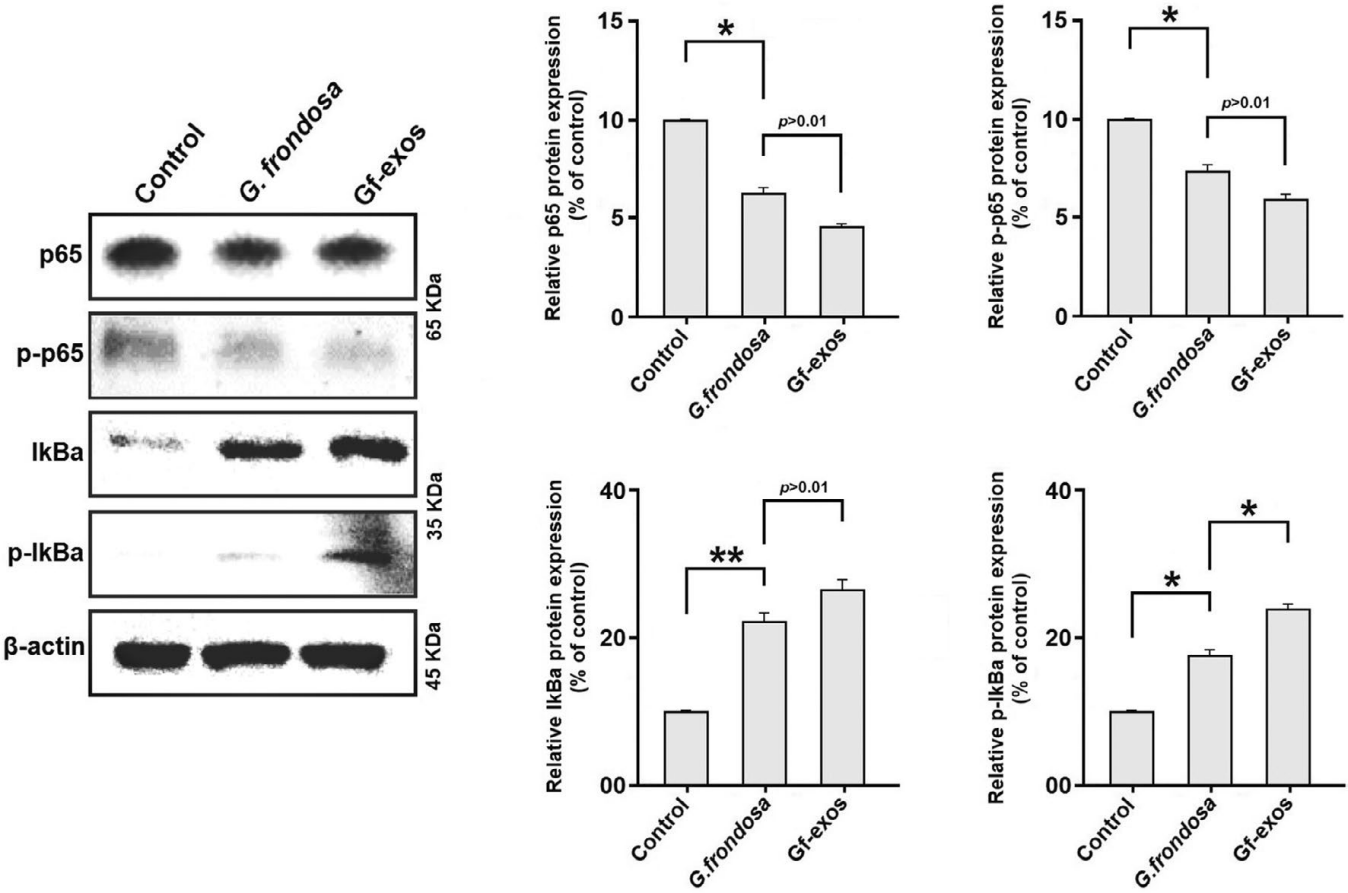
Protein expression in the NF‐κB signaling pathway (p65, p‐p65, IκB, and p‐IκB) post‐treatment. It was observed that free *G. frondosa* extraction and Gf‐exosomes significantly reduced the expression of p65, p‐p65, IκB, and p‐IκB proteins in lung cancer cells. Furthermore, Gf‐exosomes were found to dramatically modify the NF‐κB signaling pathway in cancer cells, in comparison to free *G. frondosa* extraction (*p < 0.01; **p < 0.001).

**FIGURE 12:**
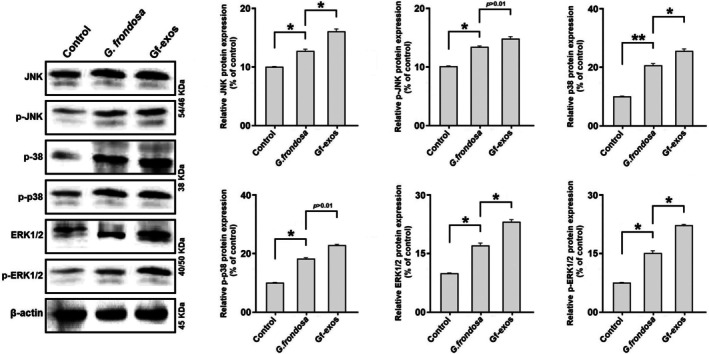
Protein expression in MAPK signaling pathways (JNK, p‐JNK, p38, p‐p38, ERK1/2, and p‐ERK1/2) post‐treatment. Treatment by free *G. frondosa* extraction and Gf‐exos significantly increased the expression of JNK, p‐JNK, p38, p‐p38, ERK1/2, and p‐ERK1/2 proteins in the lung cancer cells. The modification of the MAPK signaling pathway in the cancer cells treated by Gf‐exos was significantly more as compared with free *G. frondosa* extraction (*p < 0.01; **p < 0.001).

Western blotting was performed to assess the influence of *G. frondosa* extract and extract‐exosomes on protein levels related to the NF‐κB and MAPK signaling pathways in lung cancer cells. In the context of the NF‐κB signaling pathway, both treatments led to decreased levels of phosphorylated and total p65, while increasing the levels of phosphorylated and total IκB (Figure 11). Concerning the MAPK signaling pathway, the treatment significantly elevated the phosphorylation and total levels of p38, JNK, and ERK1/2. The results demonstrated that extract exosomes exert a more potent effect on regulating the MAPK and NF‐κB signaling pathways than the free *G. frondosa* extract (Figure 12).

## 4 Discussion

NSCLC is the primary cause of cancer‐related deaths globally, marked by a bleak prognosis and limited survival periods. A significant obstacle to NSCLC chemotherapy is the development of drug resistance, which can occur through multiple mechanisms. These include changes in drug targets, the role of microRNAs, drug inactivation, hypoxia, decreased activation of prodrugs, drug efflux, and the disruption of cell death and survival pathways (Zhou and Yang 2023; Terlizzi et al. 2019; Zhang et al. 2022). In recent times, there has been a growing interest in the use of natural compounds and advanced drug delivery systems to combat drug resistance in NSCLC (Surien et al. 2019; Reyes et al. 2024). Exosomes have emerged as a promising class of targeted delivery systems due to their biocompatibility, low cytotoxicity, and minimal immunogenicity. These vesicles can be loaded with therapeutic agents to enhance the efficacy of drugs and suppress tumor cell proliferation. For example, studies have shown that doxorubicin‐loaded exosomes derived from mesenchymal stem cells exhibit superior antitumor effects compared to free doxorubicin in osteosarcoma models (Wei et al. 2022). Similarly, exosomes loaded with 5‐fluorouracil have demonstrated enhanced anticancer effects in cholangiocarcinoma (Chen et al. 2022). However, challenges remain in mass‐producing exosomes and efficiently loading them with drugs.

This study used exosomes derived from mesenchymal stem cells loaded with *G. frondosa* extract to treat lung cancer cells. *G. frondosa* is a basidiomycete fungus from the Grifolaceae family, known for its edible fruiting bodies and exceptional anticancer properties. These properties include protecting healthy cells, preventing tumor metastasis, and inhibiting tumor growth (Guo et al. 2020, 2022; Bahar et al. 2023; Zhou and Yang 2023; Terlizzi et al. 2019; Zhang et al. 2022; Surien et al. 2019; Reyes et al. 2024; Wei et al. 2022; Chen et al. 2022; Ren et al. 2023; Kim et al. 2022; Li et al. 2024). Our research specifically focused on *G. frondosa*'s ability to induce apoptosis and autophagy through modulation of the NF‐κB and MAPK signaling pathways. Our findings indicated that *G. frondosa* reduced cell proliferation, invasion, migration, and colony formation in lung cancer cells. Additionally, the treatment increased rates of apoptosis and autophagy, leading to cell cycle arrest in sub‐G1 and G1 phases and affecting both cell and mitochondrial membrane integrity. These effects can be attributed to the modulation of key genes involved in cell viability pathways. Importantly, the use of exosomes as carriers significantly enhanced the anticancer effects of *G. frondosa* and reduced the required drug concentration compared with the free *G. frondosa* extraction form of the compound. This highlights the potential of mesenchymal stem cell‐derived exosomes as effective carriers of anticancer agents.

Although numerous studies have explored the effects of *G. frondosa* on various types of human cancers, there is a scarcity of literature specifically addressing its suppressive impact on lung cancer cells (Wu et al. 2021; Zhao, Guo, Zhang, et al. 2021; Chan et al. 2011). For example, Zhao et al. reported that when combined with vitamin C, *G. frondosa* exhibited significant antitumor and immunomodulatory effects in mouse liver cancer models by increasing apoptosis and autophagy through the modulation of apoptosis‐related factors (Zhao, Guo, Zhang, et al. 2021). Similarly, Chan et al. demonstrated that *G. frondosa* markedly decreased the viability of rat glioma C6 cells, suggesting that chemical phosphorylation could amplify its adjuvant and growth‐inhibitory properties (Chan et al. 2011). This investigation represents a significant advancement in understanding the specific effects of *G. frondosa* on lung cancer cells. A significant contributor to the high mortality rate associated with cancer is the metastasis and tumorigenesis in distant organs (Abbasi et al. 2021). The findings of this study indicate that *G. frondosa* significantly impairs the invasive and migratory capabilities of lung cancer cells, which may impede metastasis across various human cancer cells. A common objective of therapeutic strategies is to induce apoptosis and autophagy in order to combat the growth of cancerous cells (Taheri et al. 2020; Isazadeh et al. 2020).

Our findings demonstrate that treatment with *G. frondosa* significantly enhances apoptosis and autophagy in lung cancer cells. This effect is mediated by the upregulation of pro‐apoptotic genes (BAX, CASP3, CASP8, CASP9, and SMAC) and the downregulation of the anti‐apoptotic gene BCL2. Furthermore, a substantial increase in mitochondrial membrane disruption, a key step in intrinsic apoptosis, was observed in treated cells. Cell cycle analysis revealed an accumulation of cancer cells in the G1 and sub‐G1 phases and a decrease in the G1/S phase transition. This suggests that *G. frondosa* impedes cell proliferation by inducing cell cycle arrest, potentially linked to the initiation of apoptosis. It is important to note that the MAPK signaling pathway plays a crucial role in the survival, proliferation, metastasis, and invasion of most cancer cells, highlighting the potential for further investigation into *G. frondosa*'s interaction with this pathway (Guo et al. 2020). Inhibiting this pathway can enhance the effectiveness of various anticancer agents and therapies (Anjum et al. 2022).

Our study demonstrates that *G. frondosa* activates the MAPK signaling pathway in lung cancer cells. Conversely, the NF‐κB signaling pathway is crucial in cancer cell proliferation, survival, and carcinogenesis, as supported by numerous studies (Kim et al. 2022). Inhibiting the NF‐κB pathway triggers apoptosis and hinders the invasive and migratory abilities of different cancer cell types (Li et al. 2020; Gado et al. 2023). We have confirmed that *G. frondosa* significantly suppresses the NF‐κB pathway in lung cancer cells. These findings imply that the modulation of the MAPK and NF‐κB signaling pathways may be fundamental to the inhibitory effects of *G. frondosa* on the proliferation, survival, migration, and invasion of lung cancer cells.

## 5 Conclusions

This study highlights the potential of *G. frondosa* as a therapeutic agent for lung cancer. Our research demonstrates that *G. frondosa* exerts inhibitory effects on lung cancer cells by regulating key cellular processes such as apoptosis, autophagy, and critical signaling pathways. While this study shows the potential of *G. frondosa* ‐loaded exosomes for treating lung cancer, further research is necessary. It is important to understand the exact mechanisms by which *G. frondosa* affects lung cancer cells to comprehend its therapeutic capabilities fully. Our findings successfully demonstrate that *G. frondosa* extract can be incorporated into exosomes derived from stem cells. This method significantly enhances the anticancer properties of *G. frondosa* , as shown by the inhibition of lung cancer cell growth and movement through the induction of apoptosis by modifying NF‐κB and MAPK signaling. These results emphasize the potential of exosome‐based delivery for targeted therapy against lung cancer. Future clinical trials should be conducted to assess the safety and effectiveness of this innovative approach.

## Author Contributions

C.Z. and D.S.: conceptualization and consultation; contributed to methodology development and performing experiments; involved in data analysis and interpretation; contributed overall review, writing, and editing of the manuscript. All authors discussed, read, and approved the contents of the final manuscript and its revised version.

## Ethics Statement

The authors have nothing to report.

## Ethics Statement

The authors have nothing to report.

## Conflicts of Interest

The authors declare no conflicts of interest.

## Data Availability

Data are made available upon reasonable request by contacting the corresponding author.
